# Skeletal Muscle Response to Deflazacort, Dexamethasone and Methylprednisolone

**DOI:** 10.3390/cells8050406

**Published:** 2019-05-01

**Authors:** Alan Fappi, Juliana de Carvalho Neves, Leandro Nunes Sanches, Pedro Victor Massaroto e Silva, Guilherme Yuiti Sikusawa, Thayane Pereira Correa Brandão, Gerson Chadi, Edmar Zanoteli

**Affiliations:** Department of Neurology, Faculdade de Medicina FMUSP, Universidade de Sao Paulo, São Paulo, SP 01246-904, Brazil; alanfappi@usp.br (A.F.); jcneves@usp.br (J.d.C.N.); leandronsan@usp.br (L.N.S.); ppvmassa@hotmail.com (P.V.M.eS.); guilhermeys@uol.com.br (G.Y.S.); thayane.cpb@outlook.com (T.P.C.B.); gerchadi@usp.br (G.C.)

**Keywords:** glucocorticoid, skeletal muscle, muscle atrophy, IGF-1, MEK/ERK, Myostatin

## Abstract

Glucocorticoids represent some of the most prescribed drugs that are widely used in the treatment of neuromuscular diseases, but their usage leads to side effects such as muscle atrophy. However, different synthetic glucocorticoids can lead to different muscle effects, depending upon its chemical formulation. Here, we intended to demonstrate the muscle histologic and molecular effects of administering different glucocorticoids in equivalency and different dosages. **Methods**: Seventy male Wistar rats distributed into seven groups received different glucocorticoids in equivalency for ten days or saline solution. The study groups were: Control group (CT) saline solution; dexamethasone (DX) 1.25 or 2.5 mg/kg/day; methylprednisolone (MP) 6.7 or 13.3mg/kg/day; and deflazacort (DC) 10 or 20 mg/kg/day. At the end of the study, the animals were euthanized, and the tibialis anterior and gastrocnemius muscles were collected for metachromatic ATPase (Cross-sectional area (CSA) measurement), Western blotting (protein expression of IGF-1 and Ras/Raf/MEK/ERK pathways) and RT-PCR (*MYOSTATIN, MuRF-1*, *Atrogin-1, REDD-1, REDD-2, MYOD, MYOG* and *IRS1/2* genes expression) experiments. **Results**: Muscle atrophy occurred preferentially in type 2B fibers in all glucocorticoid treated groups. DC on 10 mg/kg/day was less harmful to type 2B fibers CSA than other doses and types of synthetic glucocorticoids. In type 1 fibers CSA, lower doses of DC and DX were more harmful than high doses. DX had a greater effect on the IGF-1 pathway than other glucocorticoids. MP more significantly affected P-ERK1/2 expression, muscle fiber switching (fast-to-slow), and expression of *REDD1* and *MyoD* genes than other glucocorticoids. Compared to DX and MP, DC had less of an effect on the expression of atrogenes (*MURF-1* and *Atrogin-1*) despite increased *MYOSTATIN* and decreased *IRS-2* genes expression. **Conclusions**: Different glucocorticoids appears to cause muscle atrophy affecting secondarily different signaling mechanisms. MP is more likely to affect body/muscles mass, MEK/ERK pathway and fiber type transition, DX the IGF-1 pathway and *IRS1/2* expression. DC had the smallest effect on muscle atrophic response possibly due a delayed timing on atrogenes response.

## 1. Introduction

The glucocorticoids (cortisol or hydrocortisone) are cholesterol-derived hormones that are produced in the *zona fasciculata* of the adrenal glands cortex [1]. Moreover, many studies have indicated an extra-adrenal cortisol production, for example, in the primary lymphoid organs, intestines, cardiovascular system, and central nervous system [2,3,4]. Glucocorticoid hormones serve to influence several functions, which include glycemic control, glycogen metabolism, anti-inflammatory response and immunosuppressive therapy.

Glucocorticoid treatment comprises several conditions including endocrine and non-endocrine scenarios, as well as hormonal replacement in adrenal insufficiency besides inflammatory/auto-immune and lymphoproliferative disorders [5,6]. Glucocorticoids such as Deflazacort™ are widely used as first-line treatments in Duchenne’s muscular dystrophy (DMD), bringing a better disease-course prognosis regarding motor skills, muscle strength, respiratory conditions, and cardiac function [7,8,9].

Although indicated in certain cases, glucocorticoids’ long-term use, dosage, administration route, and type lead to negative effects comprising several changes in the whole-body physiology that affects several organ systems, such as the gastrointestinal, dermatological, neurological, endocrinological, ophthalmologic, cardiovascular, and musculoskeletal systems. Regarding the musculoskeletal system, the main side effects include muscle myopathy/atrophy, osteoporosis/osteopenia, bone necrosis, pathological fracture of the long bones, tendon rupture, and muscle insulin-resistance [10,11]. It is important to point out that the glucocorticoid-induced muscle atrophy is one of the most common drug-induced myopathies, with an approximate incidence of 60% [12].

During the muscle atrophy process by glucocorticoids, there is an increased muscle degradation associated with an inhibition of muscle synthesis, leading to an atrophic state of the tissue [11]. The impact upon Akt phosphorylation leads to repression of the mTORC1 (mTOR complex, composed of mTOR, Raptor, MLST8, PRAS40 and DEPTOR) suppressing downstream proteins such as P70S6K and elf4E (related to the initiation phase of mRNA translation), and consequently reducing protein synthesis [13,14,15]. The degradation process occurs by an increased transcription of *REDD1* and *REDD2*, high activation of the Myostatin/Smad2/3 pathway [11] and—though incompletely understood—activation of the Ras/Raf/MEK/ERK pathway.

Glucocorticoid effects occur preferentially on the fast-twitch skeletal muscle, with few or no effect on the slow twitch muscle and cardiac muscle, in part explained by the PGC-1α expression [16] and differential glucocorticoid receptor (GR) expression between fiber types [17].

The atrophic state produced by glucocorticoids is resultant of autophagy-lysosome and mainly by the ubiquitin proteasome system activation [18] with a highly regulated expression of Atrophy F-box (MAFbx/Atrogin-1) and Muscle Specific Ring Finger Protein 1 (MuRF1). Atrogin-1 and MuRF-1 are E-3 ligases transcript by FOXO1/3a [18] and KLF15 [17] and are considered necessary requisites for the muscle atrophy by glucocorticoids [19,20].

Many studies have compared different glucocorticoids in a wide variety of conditions in order to better describe their responses to a given condition, which makes certain medicines more eligible in the clinical approach. Trials comparing the side effects of glucocorticoids administration are common for the DMD treatment. Since the 1980′s, after the establishment of the Clinical Investigation group of Duchenne Dystrophy (CIDD), many studies have been shown that Deflazacort leads to fewer side effects during chronic medication in comparison to prednisone (first promising test by CIDD) considering bone mineralization [21,22], and glucose tolerance [23], but with comparable effects considering the efficacy to the progression of DMD with the advantage of fewer side effects [24] despite being more likely associated to the development of cataracts [25,26]. However, some of the studies point to differences in lean body mass between people treated with different groups of glucocorticoids, even though the dystrophic pattern makes the evaluation of muscle mass gain/loss difficult; this leads to the conduct of comparative studies of the effect of glucocorticoid in a healthy system, which are scarce and few employ glucocorticoid drugs in healthy species by exploring the equivalency and cell changes, possible interventions while attenuating their side effects. The few available studies have shown opposite effects in the diaphragm and gastrocnemius muscles. Dekhuijzen et al. (1995) [27] showed that in rats, Deflazacort, more so than methylprednisolone (equivalent doses), was harmful to the areas of peripheral muscles as well as the diaphragm. Anderson et al. (1996) have found that Deflazacort but not prednisone improves both muscle repair and fiber growth in the diaphragm [28].

Considering the lack of comparative analysis of glucocorticoid-induced muscle atrophy in healthy individuals, this work aimed to evaluate the impact of three main classes of glucocorticoid drugs (dexamethasone, methylprednisolone, and deflazacort) in two doses, in terms of pharmaceutical equivalency, on healthy Wistar rats with respect to the muscle fiber size, proportion, and cell trophism signaling.

## 2. Material and Methods

### 2.1. Animals and Drug Treatment

Male Wistar rats (70 animals) between 10 and 12 weeks of age and weighing between 320 to 350 g were used. The animals were housed in cages containing no more than three individuals under dark-light cycles for 12 h each at 25 °C and received food and water ad libitum. The animals were provided with commercial feed (Nuvital, Nuvilab CR-1, NUVITAL Nutrientes LTDA, Curitiba, Brazil) containing crude protein (min 22.0%), ethereal extract (min 4.5%), mineral matter (max 1 d 0.0%), fibrous matter (max 8.0%), calcium (max 1.4%), phosphor (min 0.8%), vitamin A (25,200.00 UI/kg), vitamin D3 (2100.00 UI/kg), vitamin E (60.00 mg/kg), vitamin K3 (12.50 mg/kg), vitamin B1 (14.40 mg/kg), vitamin B2 (11.00 mg/kg), vitamin B6 (12.00 mg/kg), vitamin B12 (60.00 mcg/kg), niacin (60.00 mg/kg), pantothenic acid (112.00 mg/kg), folic acid (6.00 mg/kg), biotin (0.26 mg/kg), choline (1100.00 mg/kg), iron (50.00 mg/kg), zinc (60.00 mg/kg), copper (10.00 mg/kg), iodine (2.00 mg/kg), manganese (60.00 mg/kg), selenium (0.05 mg/kg), cobalt (1.50 mg/kg), lysine (100.00 mg/kg), methionine (300.00 mg/kg), and antioxidant (100.00 mg/kg). All in vivo experiments were approved by our local research ethics committee (CEUA FMUSP, process 430/2013) and were performed according to the NIH guidelines on the care, handling, and use of laboratory animals.

### 2.2. Glucocorticoid Administration

Dexamethasone doses were established according to previously used and well defined doses able to induce significant muscle atrophy in this model [29,30] and the other glucocorticoids classes were adjusted to its dose equivalency according to the available literature [31,32].

Initially, all 70 animals were kept in their cages for a 30-day period with periodic body weight checks. After this period, the animals received one of the following glucocorticoid doses administered via the designated pathway: Dexamethasone (injectable Decadron^®^ 4mg/mL, Áche laboratories, São Paulo, Brazil) subcutaneously (s.c.) injected in the dorsal region at doses of 2.5 mg/kg/day or 1.25 mg/kg/day for 10 days; Methylprednisolone (methylprednisolone acetate, injectable suspension, Depo-Medrol^®^ 40 mg/mL, Pfizer, New York, NY, USA) via intramuscular (i.m.) injection into the hamstring region at doses of 13.3 mg/kg/day or 6.7 mg/kg/day for 10 days; and Deflazacort (manufactured oral suspension, 40 mg/mL, Rhamus Laboratory, São Paulo, Brazil) via gavage (v.g.) at doses of 20 mg/kg/day or 10 mg/kg/day for 10 days. The control group (CT) received only saline solution (0.9% NaCl in ultrapure water) delivered via intramuscular injection into the hamstrings for 10 days. The injections were applied at approximately 1 p.m. alternating sides and locality to prevent wounds and avoid animal stress.

At the end of the experiment, seven study groups were established: (1) the CT group (i.m. saline solution administration for 10 days), (2) the DX1.25 group (s.c. 1.25 mg/kg/day of dexamethasone administration for 10 days), (3) the DX2.5 group (s.c. 2.5 mg/kg/day of dexamethasone administration for 10 days), (4) the MP13 group (i.m. 13.3 mg/kg/day of methylprednisolone administration for 10 days), (5) the MP6 group (i.m. 6.7 mg/kg/day of methylprednisolone administration for 10 days), (6) the DC20 group (10 days of v.g. 20 mg/kg/day of deflazacort administration), and (7) the DC10 group (v.g. 10 mg/kg/day of deflazacort administration for 10 days).

After 10 days of glucocorticoids treatment, the animals were euthanized with an intraperitoneal injection of sodium pentobarbital (30 mg/kg) and their gastrocnemius (GA) and tibialis anterior (TA) muscles and adrenal glands (AGs) were immediately dissected, weighed, snap frozen in isopentane cooled in liquid nitrogen (muscles collected for histology), and stored at −80 °C.

### 2.3. Cross-Sectional Areas Evaluation

TA cross-sections were performed in cryostat (Leica, model CM3000, Leica Biosystems, Wetzlar, Germany) at −25 °C. The sections were submitted to the metachromatic dye-ATPase method (mATPase) according to Ogilvie and Feeback [33] to differentiate the main muscle fiber subtypes (e.g., 1, 2A, and 2B). Slides were photographed at 20X (Olympus, microscope AX70, camera and software Olympus DP72), and the cross-sectional areas (CSA) were measured for each muscle fiber subtype using the analysis tool in the Photoshop CS6 extended software. Prior to the measurements, a pixel-to-micrometer conversion scale was established in the Image J software (java 8 for Windows, NIH, Bethesda, MD, USA) based on pictures of a micrometer slide at the same magnification (20X). An average of 350 fibers of each muscle fragment (animal) was measured.

### 2.4. SDS-PAGE (Western Blotting)

TA fragments were homogenized with cooled RIPA buffer (PBS pH 7.4, 0.5% sodium deoxycholate, 0.1% SDS, 1 mM EDTA pH 8.0, 1 mM EGTA pH 8.0, 50 mM Tris-Hcl, 1% NP-40, 10 mM NaOV, 10 mM NaPyr, 50 mM NaF, and 1% protease inhibitor Sigma P8340) in 20X *w*/*v* and centrifuged for 5 min at 4 °C at 16,100 g. The supernatants were quantified using Bradford reagent (Bio-Rad, #500-0006, Hercules, CA, USA) and the BSA standard curve. The samples were boiled at 100 °C for 5 min and then applied to 8 or 10% bis-acrylamide mini-gels, within 50 to 80 µg protein load per well. In sequence, the samples were transferred to PVDF or nitrocellulose membranes at 65 V for 1 h in a Criterion Blotter (Bio-Rad) apparatus. The membranes were blocked in 5% BSA for 1 h and incubated overnight with primary antibody (1:1000) diluted in a blocking solution. Anti-rabbit HRP-conjugated secondary antibody (GE Healthcare Bio-Sciences, #NA934, Pittsburgh, PA, USA) [1:10,000] diluted in a blocking solution (5% BSA in TBS-T) was incubated for 1 h at room temperature and then the ECL (Enhanced Chemiluminescent) reagent (Millipore/Sigma, #WBKLS0500, Burlington, MA, USA) was incubated for 5 min, prior to scanning in the C-DiGit Blot Scanner (LI-COR Biosciences, Lincoln, NE, USA) for 12 min. For protein loading control, labeling densities were normalized by membrane staining against total protein across the lane (250–10 kDa) of the correspondent sample. Line blots of approximate weight “total protein” are displayed in each corresponding graph for illustration. Blots were analyzed with the Image Studio software version 4.0 (LI-COR).

The primary antibodies used included anti-Akt pan (Cell Signaling Technology, #4691, Danvers, MA, USA), anti-P-Akt (Ser473) (Cell Signaling, #4060), anti-GSK-3β (Cell Signaling, #9315), anti-P-GSK-3β (Ser9) (Cell Signaling, #9322), anti-FOXO3a (Cell Signaling, #2497), anti-P-FOXO3a (Ser253) (Cell Signaling, #9466), anti-ERK1/2 (Cell Signaling, #4695), and anti-P-ERK1/2 (Thr202/Tyr204) (Cell Signaling, #4377).

### 2.5. Quantitative PCR

The total RNA was extracted from 30 mg of GA muscles using the SV Total RNA Isolation System (Promega, #Z3105, Madison, WI, USA) according to the guidelines for the preparation of lysates from small tissue samples. RNA pellets were resuspended in 50 µL nuclease-free water for a final RNA concentration of 500 ng/µL. RNA purity and integrity were tested by the spectrophotometry and agarose gel, respectively. In sequence, reverse transcription reactions were performed using the GoScript Reverse Transcription Mix Kit (Promega, #A2801) following the manufacturer’s instructions. Quantitative PCR was done in duplicate with a GoTaq qPCR Master Mix (Promega, #A6002) loading [75 ng] of cDNA and 50 nM of sense and antisense primers in a Piko Real 96 (Thermo Scientific, TCR0096, Waltham, MA, USA) equipment. The data obtained were systematized and analyzed according to the calculation of 2^−∆∆*C*T^.

Primer sequences: MURF-1 forward TCGACATCTACAAGCAGGAA, reverse CTGTCCTTGGAAGATGCTTT; Atrogin-1/MAFbx forward TGAAGACCGGCTACTGTGGAAGAGAC, reverse TTGGGGTGAAAGTGAGACGGAGCAG; REDD-1 forward CACCGGCTTCAGAGTCATCA, reverse CGGGTCTCCACCACAGAAAT; REDD-2 forward CTTCAGCGTCTGGTGAAATCC, reverse ATGCTGGCCGTGTTCTTACTG; IRS-1 forward CCCGGTCGGTGCCAAATAGC, reverse GCCACTGGTGAGGTATCCACATAGC; IRS-2 forward CCACACACCTGTCCTCATTG, reverse TAATCCGCTTTGCCAAAATC; GAPDH forward ACGCCAGTAGACTCCACGAC, reverse ATGACTCTACCCACGGCAAG.

### 2.6. Statistical Analysis

The quantitative results were analyzed in the GraphPad Prism 5.0 software (GraphPad Software, San Diego, CA, USA). They were initially classified according to normality using the Shapiro-Wilk normality test and then evaluated with the appropriate statistical tests. The differences between means were analyzed using the unpaired Student’s t-test, and differences among groups were analyzed by one-way ANOVA followed by the Bonferroni post hoc test. The test used, and the number of individuals per group, are described in the presentation of each result. Variations with *P* < 0.05 were considered statistically significant.

## 3. Results

### 3.1. Body and Tissue Weights Resulting from Different Glucocorticoids Administration

**Figure 1:**
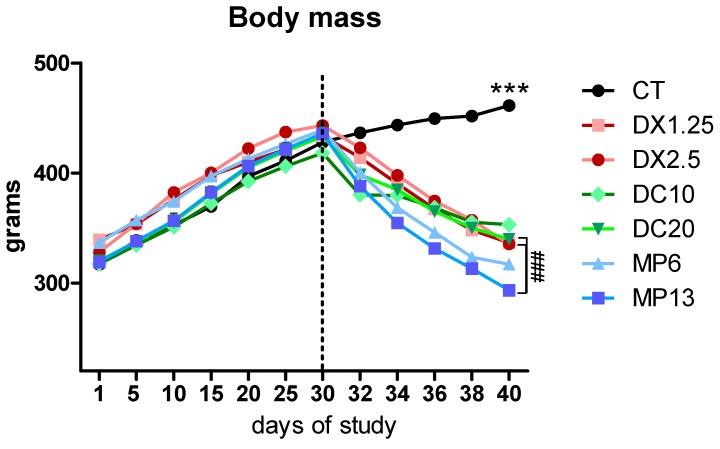
Body mass during the study. The dotted line represents the initial moment of glucocorticoid administration. Asterisks represent statistical difference between the control groups (CT) vs. all groups (#) represent statistical difference between groups. Data are presented as mean. *** or ### = *P* < 0.001.

There was a linear, progressive and equivalent weight gain of all animals up to the beginning of drug administration (Figure 1). From the second to the 10th day of glucocorticoids’ administration, a significant decrease in the body weight of the animals on the glucocorticoid treatment was observed in relation to the control group (*P* < 0.001 for all groups) at the end of the study (Table 1). It was observed that the mean body weight of animals receiving MP at the 13.3 mg/kg/day dose was significantly lower than the groups receiving DX at 2.5 mg/kg/day and DC at 20 mg/kg/day (*P* < 0.01 in both comparisons). Comparing pre- and post-glucocorticoid administration, the DX1.25 group had a body weight loss of 22.4%, the other group results were DX2.5, 24.31%; DC10, 15.59%; DC20, 21.56%; MP6, 27.79%; and MP13, 32.70%.

The results of the GA and TA weights confirm the body weight curve data with a great loss of muscle weight in the groups that received glucocorticoids in relation to the CT group (*P* < 0.001 in all comparisons) (Table 2). The MP13 group showed a significantly greater muscle weight loss of GA compared to the DX2.5 and DC20 groups (*P* < 0.05 and *P* < 0.01, respectively), as well as in relation to the MP6 group (*P* < 0.001). The MP13 group had less TA muscle weight compared to the DC20 group (*P* < 0.05).

Considering the adrenal weight measurement (an indirect evaluation of HPA axis) DC10 or MP6 did not cause a significant adrenal weight loss in comparison to the CT group, as observed after the DX administration in both doses with a weight loss of approximately 51% (*P* < 0.001 to both doses in comparison to the CT group).

These data show how DX caused more negative effects to the HPA axis, even in lowest equivalent dose compared to the MP or DC; however, higher MP was more harmful to muscles or body weight than other evaluated glucocorticoids.

### 3.2. Cross-Sectional Areas (CSA) and Fiber Type Proportion Resulting from Different Glucocorticoids Administration

**Figure 2:**
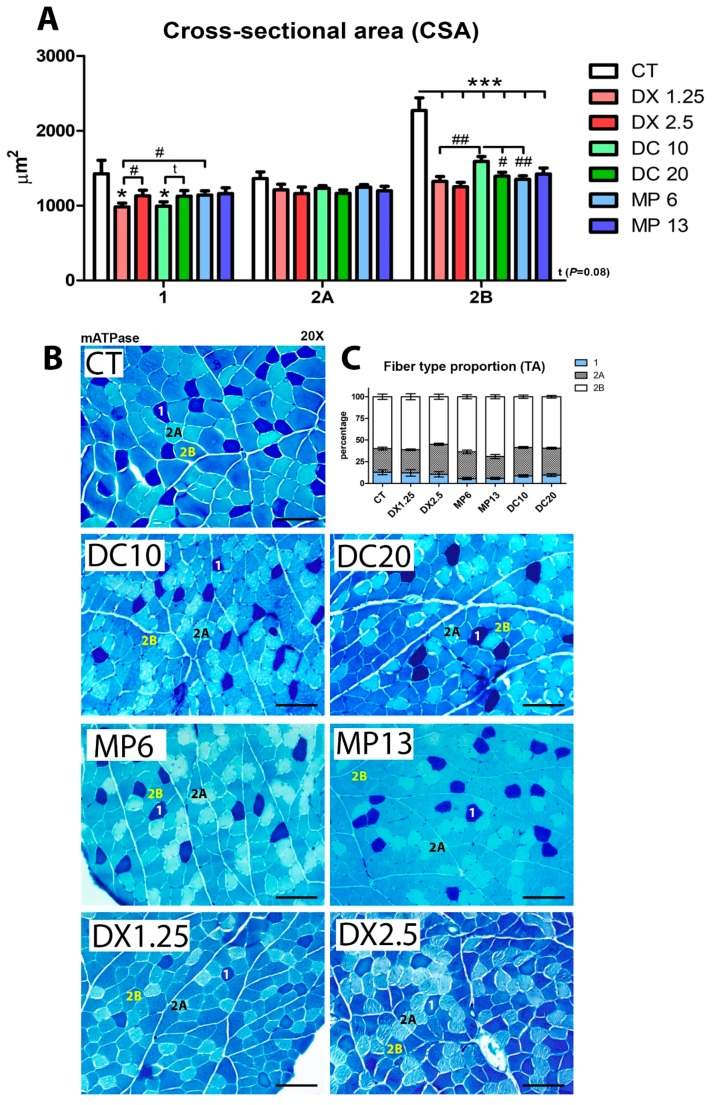
Muscle cross-sectional area and muscle fiber type proportions analyzed after the administration of different glucocorticoids. (**A**) Cross-sectional area—(*) represents statistical difference in comparison to the control group (CT), and (#) represents statistical difference between two groups. Legend: * or # = *P* < 0.05; ## = *P* < 0.01 and *** = *P* < 0.001 (*n* per group: CT = 10, DX1.25 = 9, DX2.5 = 8, DC10 = 10, DC20 = 10, MP13 = 8, MP6 = 8). One-way ANOVA, followed by the Bonferroni post hoc test, was used for each fiber type analysis; (**B**) histological mATPase photographs, scale bar = 100 µm; (**C**) muscle fiber type proportions (statistical analysis is expressed in Table 3).

The mATPase method was used to differentiate types 1, 2A, and 2B muscle fibers, in order to evaluate how different glucocorticoids would influence CSA after 10 days of administration (Figure 2A,B).

The CSA of type 1 fibers decreased only on the DX1.25 and DC10 groups (fewer doses) in comparison to the CT (*P* < 0.05 to both) and in comparison to the higher dose groups DX2.5 (*P* < 0.05) and DC20 (*P* = 0.09). MP at both doses did not cause significant changes in type 1 muscle fibers.

None of the glucocorticoids in either doses led to changes in 2A muscle fibers in comparison to the CT.

In type 2B fibers, a significant CSA decrease was observed in all groups in comparison to the CT group (*P* < 0.001 for all). However, among the glucocorticoid groups, there was an important variation: Animals of the DC10 group had a higher CSA of 2B fibers in comparison to the higher dose group, DC20 (*P* < 0.05), and the equipotent doses groups, DX1.25 and MP6 (*P* < 0.01 for both).

In order to evaluate the muscle fiber type transition after 10 days of different glucocorticoid administration, we calculated the proportion of muscle fibers (Figure 2C) classified from a pool of 500 muscle fibers per animal.

We observed a significant decrease in the proportion of type 1 muscle fibers in both groups receiving MP (MP13 and MP6) in relation to the CT group (*P* < 0.01 and *P* < 0.05, respectively). The proportion of type 1 fibers in the MP6 group was significantly lower than in the equipotent dose groups DX1.25 and DC10 (*P* < 0.05 for both).

In type 2A muscle fibers, there was a significant increased proportion in the DX2.5 (*P* < 0.01) and DC10 (*P* < 0.05) groups compared to the CT group. A significant difference was found when comparing the DX2.5 group to the DX1.25 group (*P* < 0.01). It was observed that the MP13 group presented the lowest proportion of 2A fibers in relation to the other groups, DX2.5 (*P* < 0.01) and DC20 (*P* < 0.05).

In type 2B muscle fibers, the MP13 group showed the highest proportion of fibers in relation to the CT group (*P* < 0.05), and compared to the other groups, DX2.5 (*P* < 0.01) and DC20 (*P* < 0.01).

These data show that MP at both doses significantly affects the muscle fiber switching program (slow-to-fast) during muscle atrophy more than other glucocorticoids. Additionally, DX and DC at lower doses are more harmful to the type 1 fiber CSA than higher doses.

### 3.3. IGF-1 and Atrogenes Expression Resulting from Different Glucocorticoids Administration

**Figure 3:**
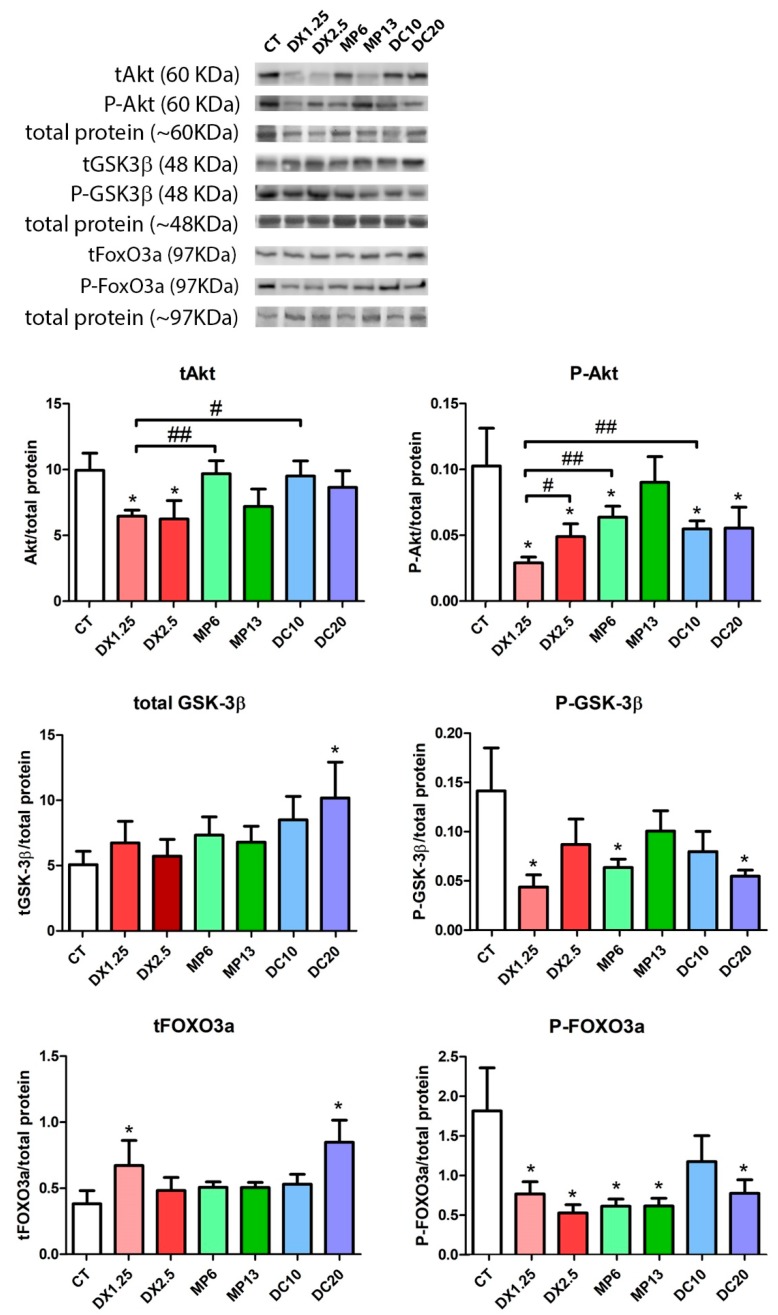
Western blotting analysis of the IGF-1 pathway after administration of different glucocorticoids. (*) represents the statistical difference in comparison to the CT and (#) represents statistical difference between groups. Legend: * or # = *P* < 0.05; ## = *P* < 0.01 (*n* per group: CT = 10, DX1.25 = 9, DX2.5 = 8, DC10 = 10, DC20 = 10, MP13 = 8, MP6 = 8).

**Figure 4:**
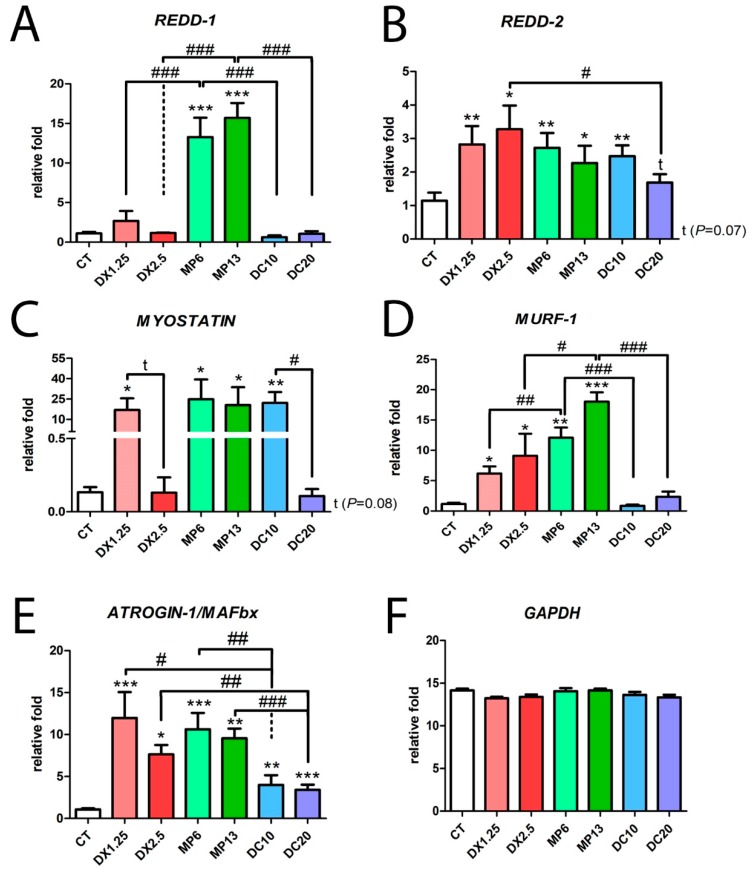
mRNA expression by RT-PCR of *MURF-1*, *Atrogin*-*1/MAFbx*, *REDD-1*, *REDD-2* and *MYOSTATIN* after administration of different glucocorticoids. Atrogenes expression (**A**–**E**), Myostatin expression (**C**), housekeeping gene expression GAPDH (**F**). (*) represents the statistical difference in comparison to the CT, and (#) represents statistical difference between groups. Legend: * or # = *P* < 0.05; ** or ## = *P* < 0.01 and *** or ### = *P* < 0.001. (*n* per group: CT = 10, DX1.25 = 9, DX2.5 = 8, DC10 = 10, DC20 = 10, MP13 = 8, MP6 = 8).

Considering the importance of the IGF-1/PI-3k/Akt/mTOR pathway in the muscle atrophy process, we analyzed its three main components: Total and phosphorylated forms, (Akt, GSK-3β, and FoxO3a) protein expression by Western blotting (Figure 3), and atrogenes mRNA expression (related to the FoxO transcriptional activity and ubiquitin-proteasome system) by quantitative PCR (Figure 4).

We observed that the expression of total Akt in animals receiving DX1.25 and 2.5 was significantly lower in comparison to the CT group (*P* < 0.05 for both) and, in comparison, to the DC10 (*P* < 0.05) and MP6 (*P* < 0.01) groups. No differences were observed between the higher dose groups compared to the lower dose groups receiving DX. In the expression of P-Akt, a significant decrease was observed in all groups in relation to the CT group, except the MP13 group, with a significant decrease in the DX1.25 group compared to other glucocorticoid groups.

The expression of total GSK3β showed that only animals from the DC20 group had a significant increase in comparison to the CT group (*P* < 0.05). The expression of P-GSK3β (Ser9) significantly decreased in the DX1.25, MP6, and DC20 groups in comparison to the CT group (*P* < 0.05 for all). No differences among glucocorticoid groups were found in GSK3 β and P-GSK3 β expressions.

The expression of total FOXO3a showed a significant increased expression in the DC20 and DX1.25 groups in comparison to the CT group (*P* < 0.05). A significant decrease in the P-FOXO3a (Ser253) expression was observed in all groups compared to the CT group, except in the DC10 group. No difference was observed among glucocorticoid groups.

The expression of *REDD-1* was significantly increased only in the groups receiving MP (the MP6 and MP13 groups) compared to the CT group and DX1.25 and DX2.5 groups (*P* < 0.001 in all comparisons) (Figure 4A). Nevertheless, the expression of *REDD-2* (Figure 4B) was significantly increased in all groups, but with a tendency to increase in the DC20 group (*P* = 0.07), compared to the CT group. For the REDD-2 expression, there was a significant difference between the groups DX2.5 and DC20 (*P* < 0.05).

The *MYOSTATIN* expression significantly increased in all groups, except in the DX2.5 and DC20 groups, in comparison to the CT group (Figure 4C). There was a significantly higher expression comparing the DC10 and DC20 groups (*P* < 0.05).

The *MuRF-1* expression significantly increased in all groups receiving DX and MP in comparison to the CT group, without changes in the DC10 or DC20 group (Figure 4D). There was a significant increase in the *MuRF-1* expression in the MP13 group when compared to the DX2.5 (*P* < 0.05) and DC20 (*P* < 0.001) groups, as well as comparing the MP6 group and the equipotent dose groups (DX1.25, *P* < 0.01 and DC10, *P* < 0.001).

The *Atrogin-1* expression (Figure 4E) significantly increased in all groups in comparison to the CT group. The DC10 and DC20 groups showed the lowest *Atrogin-1* expression in comparison to the other glucocorticoids: DC10 vs. MP6 (*P* < 0.01) and vs. DX1.25 (*P* < 0.05), DC20 vs. MP13 (*P* < 0.001) and vs. DX2.5 (*P* < 0.01).

These data showed that DX has a greater effect on the AKT total and phosphorylated state, and MP displayed higher atrogenes (*REDD-1* and *MURF-1*) expression in comparison to the other glucocorticoids.

### 3.4. Insulin Receptor Substrate 1/2 Gene Expression Resulting from Different Glucocorticoids Administration

**Figure 5:**
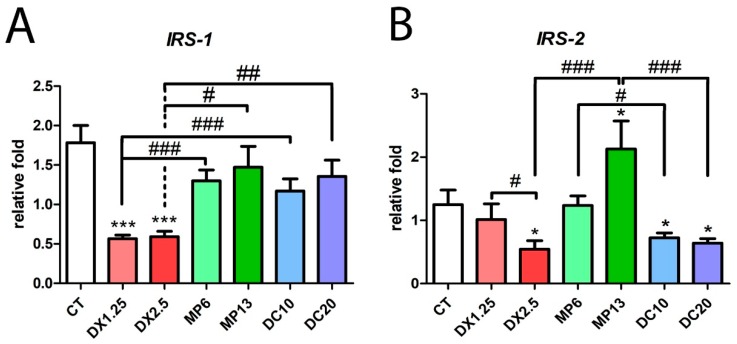
mRNA expression by RT-PCR of *IRS-1* (**A**) and *IRS-2* (**B**) after the administration of different glucocorticoids. (*) represents the statistical difference in comparison to the CT and (#) represents statistical difference between groups. Legend: * or # = *P* < 0.05; ## = *P* < 0.01 and *** or ### = *P* < 0.001. (*n* per group: CT = 10, DX1.25 = 9, DX2.5 = 8, DC10 = 10, DC20 = 10, MP13 = 8, MP6 = 8).

In an exploratory way, we evaluated the expression of insulin receptor substrates (IRS-1 and IRS-2) because of their relation to the response of several hormones and cytokines receptors such as IL-4-R, IL-9-R, IFNƴ-R, GH-R, IFNα as well as the insulin and IGF-1 receptor [34] (Figure 5).

*IRS-1* mRNA expression significantly decreased in the DX1.25 and DX2.5 groups in comparison to the CT group (*P* < 0.001 to both) and to other glucocorticoids. *IRS-2* expression significantly decreased only in the groups DX2.5, DC10, and DC20 in comparison to the CT group (*P* < 0.05 for all), and between the DX1.25 and DX2.5 groups (*P* < 0.05). Interestingly, the MP13 group had an increased *IRS-2* in comparison to the CT group (*P* < 0.05) and equipotent glucocorticoids DX2.5 and DC20 (*P* < 0.001 to both).

These data show that 10 days of DX administration is more likely to affect multiple inflammatory, myogenic and insulin response pathways than other glucocorticoids in both dosages.

### 3.5. Methylprednisolone Administration Leads to An Inversion of Myogenic mRNA Expression

**Figure 6:**
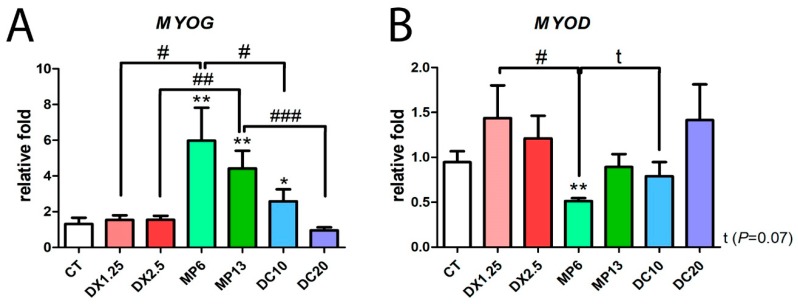
mRNA expression by RT-PCR of *MyoG* (**A**) and *MyoD* (**B**) after the administration of different glucocorticoids. (*) represents the statistical difference in comparison to the CT, and (#) represents statistical difference between groups. Legend: * or # = *P* < 0.05; ** or ## = *P* < 0.01 and ### = *P* < 0.001. (*n* per group: CT = 10, DX1.25 = 9, DX2.5 = 8, DC10 = 10, DC20 = 10, MP13 = 8, MP6 = 8).

Considering the impact of glucocorticoids upon the myogenic program, we evaluated the mRNA expression of *MyoG* and *MyoD* by a quantitative PCR (Figure 6).

Between glucocorticoids, *MyoG* expression was increased only in the groups receiving methylprednisolone (MP6 and MP13) and in the DC10 group compared to the CT group (Figure 6A). This increase in the expression of *MyoG* in the MP6 and MP13 groups was significant in comparison to the other groups: MP6 vs. DX1.25 and DC10 (*P* < 0.05 for both); MP13 vs. DX2.5 (*P* < 0.01) and vs. DC20 (*P* < 0.001).

The expression of *MyoD* decreased only in the MP6 group in comparison to the CT group (*P* < 0.01) and in the other groups with equipotent dose, DX1.25 and DC10 (*P* < 0.05 for both) (Figure 6B).

These data showed that MP administration leads to an inversion of myogenic mRNA expression during the atrophic process.

### 3.6. MEK/ERK Pathway Changes Resulting from Different Glucocorticoids Administration

We aimed to identify the influence of different glucocorticoids on the expression of ERK components once there is a possible positive effect of the Ras/Raf/MEK/ERK pathway on muscle fiber type switching [35]. Therefore, a negative regulation of MEK/ERK would cause major changes in fiber switching (fast-to-slow).

**Figure 7:**
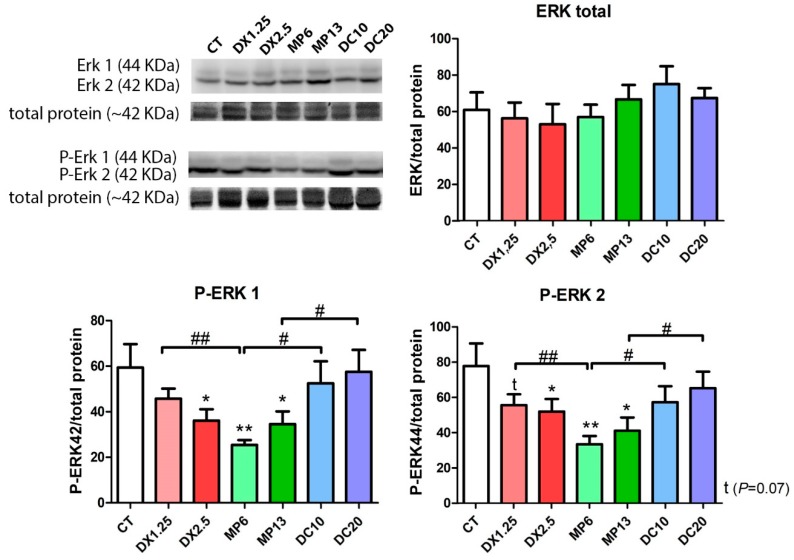
Western blotting analysis of the Ras/Raf/MEK/ERK pathway after the administration of different glucocorticoids. (*) represents the statistical difference in comparison to the CT and (#) represents statistical difference between groups. Legend: * or # = *P* < 0.05; ** or ## = *P* < 0.01 (n per group: CT = 10, DX1.25 = 9, DX2.5 = 8, DC10 = 10, DC20 = 10, MP13 = 8, MP6 = 8).

No differences regarding the expression of the total ERK1/2 was observed between the groups (Figure 7). Considering the phosphorylated forms of ERK1/2 (Thr202/Tyr204), a significant decrease of the P-ERK1 and P-ERK2 expression was observed in the DX2.5, MP6, and MP13 groups in comparison to the CT group. There was a significantly lower expression in the MP6 group in comparison to equipotent doses within the DX1.25 and DC10 groups (*P* < 0.01 and *P* < 0.05, respectively) in both P-ERK1/2 expressions.

These data showed that MP significantly affects P-ERK1/2 signalization more than other glucocorticoids, and it is possibly related to fiber type transition.

## 4. Discussion

Our study evaluated the impacts of different commonly used glucocorticoids upon healthy skeletal muscle by evaluating the cross-sectional areas of the fibers, muscle fiber type transition, main pathways related to the muscle trophism, and genes related to the muscle atrophy.

The doses of DX administered (2.5 and 1.25 mg/kg/day) consistently caused body weight loss, as well as muscle and adrenal weight decrease as expected and showed by others [29]. However, compared to the other glucocorticoids, we observed that MP at a dosage of 13 mg/kg/day was more harmful to the body and muscle weight compared to DX and DC. At a low dose comparison, the study of Dekhuijzen et al. (1995) showed that DC can cause more damage to skeletal muscle and body weight than MP in animals receiving both at low doses (0.5 mg/kg/day for six weeks) [27]. Otherwise, as observed in the study by Nava, et al. [36], high doses of MP (80 mg/kg/day) significantly affects body weight (on the 3rd day) and muscle at the end of five days of administration. Therefore, there is a dose-influence of both glucocorticoid types, deflazacort and methylprednisolone, administered on the muscle tissue. In the first study [27] was observed that the DC, in addition to the decrease in body/muscle weight, causes higher muscular atrophy in fibers types 1, 2A, and 2B in comparison to the MP and control animals. The authors used a dose of 0.4 mg/kg/day of deflazacort for six weeks, a dose several times lesser than used on this study (20 and 10 mg/kg/day).

The study by Yoshimura, et al. [37], revealed an extensive atrophy of several skeletal muscles types in healthy beagles after a low dose administration (0.9 mg/kg/day) of DC (for three months). In our study, a dose of 10 mg/kg/day caused less pre- and post-drug weight loss among glucocorticoids (15.5%); however, it caused a great muscular atrophy impact in type 1 fibers (CSA) in relation to its comparative group (DC20) and MP at 6.7 mg/kg/day. The muscle atrophy observed in type 1 fibers in animals receiving lower doses of DC or DX, rather than at twice the dose, was unexpected. However, studies using different doses of glucocorticoids also observed greater muscular atrophy (diaphragmatic) in animals receiving lower doses of glucocorticoid in comparison to those receiving higher doses. Maes et al. (2008), showed that the administration of MP in doses between 30 and 80 mg/kg/day for 24 h in rats caused attenuation of the effects of diaphragmatic dysfunction during mechanical ventilation without impact on the fiber area [38]. However, the use of a lower dose (5 mg/kg/day) led to a significant impairment of the diaphragmatic muscle [39]. The authors postulated that high-dose glucocorticoid would be able to inhibit calpain activity, reducing protein degradation and, thus, protecting the fiber loss of the cross-sectional area, which was not measured in this study, but that could certainly explain the variable dose-dependency of the muscle atrophy by the different glucocorticoids available.

Considering that there was no excessive body weight loss that could justify a possible influence in the CSA results of DC group, it was clear that the DC in smaller doses has less of an impact on type 2B fibers than MP and DX, on the other hand with more effect on type 1 fibers (as also observed in the lower-dose DX group). This finding might be related to the supposed calpain inhibition in high doses, as postulated before; however, with a plausible “fiber-specificity” effect since it was only observed in type 1 fibers in our study and in the diaphragmatic muscles by other authors [38,39], which is a musculature with a great proportion of oxidative fibers.

Regarding the adrenal gland weight, DC at a dose of 10 mg/kg/day and MP at a dose of 6.7 mg/kg/day for 10 days did not negatively influence the adrenal gland weight as observed with DX at both doses. Therefore, in short periods DX is more harmful to the trophism of the adrenal gland (HPA axis) than other glucocorticoids at the same dose equivalency, possibly due to stronger inhibitory activity under the HPA axis, corticotrophin releasing hormone (CRH) and hence adrenocorticotropic hormone (ACTH) [40,41].

At higher doses both drugs, methylprednisolone and deflazacort, similarly influenced adrenal weight when compared to DX. In the study by Dekhuijzen et al. (1995) [27], a low-dose administration of DC (0.5 mg/kg/day) or MP at a dose of 0.4 mg/kg/day for a longer period (six weeks) was sufficient to significantly decrease the weight of the rats’ adrenal gland. This indicates that low doses of both of the glucocorticoids do not influence adrenal trophism following administration of up to 10 days, but it can significantly affect their trophism if administered chronically in rats.

Nava et al. (1996), observed a significant decrease in the adrenal gland weight (~25 % decrease) of rats receiving MP at a dose of 80 mg/kg/day for seven days [36]. However, in our study, we observed a 47.8% decrease in the adrenal weight of the MP13 group compared to the CT group, almost twice of that observed by the authors using a much lower dose. This outcome points to a possible “protective mechanism” by the HPA axis at an extremely high dose of glucocorticoid, possibly by receptor sensitivity regulation, transcription, post-translational modification, or feedback.

It is known that Akt, once activated by IGF-1 by phosphorylation, leads to the activation/inhibition of several proteins, acting on the activation of mTOR, to increasing protein synthesis, and the inhibition of GSK3β, related to glucose metabolism and insulin action, which detracts from muscle synthesis [42,43,44,45,46].

Unlike other studies, our research indicated that the DX glucocorticoid was able to cause a significant decrease in the total Akt expression. However, considering AKT phosphorylation, all glucocorticoids decreased its expression, along with FoxO3a phosphorylation. Such outcomes support the literature affirming that glucocorticoids directly affect the IGF-1 pathway, causing inhibitory effects on the phosphorylation of Akt, which leads to a decreased protein synthesis and increased protein degradation [11,47]. Considering the glucocorticoid effects on inflammatory response inhibition as well as the IGF-1 and glucose uptake (peripheral insulin resistance), the *IRS-1* mRNA expression was only affected by dexamethasone (1.25 and 2.5 mg/kg/day for 10 days), probably leading to a significant impairment of insulin signaling, although we have not evaluated IRS1/2 protein expression, insulin production, glycaemia, or muscle glucose uptake. Studies have already shown that DX is able to decrease IRS-1 signaling through competition by the active glucocorticoid receptor for the P85α (substrate of the insulin receptor) impairing insulin, IGF-1 and ERK signaling [48,49].

The REDD1/2 (regulated in development and DNA damage responses 1/2) is a component related to the stress response and is activated by the use of glucocorticoids and reactive oxygen species [50,51,52]. However, the oxidative stress produces atrophy principally via activation of p38 mitogen activated protein kinase (MAPK), which subsequently induces the expression of MAFbx, MuRF1 and autophagy-lysosome system [18,53]. Studies have shown that there is a time-dependent muscle response of *REDD-1* and *REDD-2* after glucocorticoid exposure [54,55,56,57], where the *REDD-1* expression is initially elevated (first 24-48 s), but normalizes after few days accompanied by an increased *REDD-2* expression. Recapitulating the observations in our study, all groups of animals receiving any drug/dose showed an elevated *REDD-2* expression, however, only animals receiving MP (both doses) had significantly increased expressions of *REDD-1* in comparison to the other groups of glucocorticoid.

One of the inhibitory effects upon mTORC1 activity occurs through the action of REDD1 and REDD2, produced by the transcription factor FoxO3, that inhibits the action of the Rheb protein on mTOR activation affecting muscle protein synthesis [56,58]. Interestingly, Frost, et al. [59], have shown that the IGF-1 administration (in vitro and in vivo) caused an increased REDD1 (mRNA and protein) expression without affecting protein synthesis, yet, this finding has not yet been confirmed. Similarly, the animals from the MP group displayed an increased *REDD1* expression associated with the high *MyoG* expression (late myogenic program) and decreased *MyoD* (early myogenic program), and this outcome is not elucidated. Although the protein synthesis are remarkably affected by glucocorticoids [11,18,60], our efforts were directed toward the muscle atrophy impact and muscle synthesis-related proteins (e.g., P70^S6K^, EIFs, 4EBP1 and mTOR) were not evaluated, but certainly would clarify the methylprednisolone effect on both mRNA and protein related to protein synthesis in comparison to other glucocorticoids.

The same studies have shown that glucocorticoids have an effect of decreasing the muscle Ca^2+^ influx in a lesser or greater degree, depending on the type of glucocorticoid [61]. Despite a lack of studies comparing the Ca^2+^ influx between these glucocorticoids, it is known that calcium levels can positively influence the Ras/Raf/MEK/ERK pathway signaling [62]. Based on this understanding, we believe that the decrease in the P-ERK1/2 expression observed in the administration of the MP might have occurred due to alterations in the level of sarcoplasmic calcium. Such an effect would cause greater damage to an early myogenic program compared to other glucocorticoids and changes of muscle fiber proportion because ERK1/2 is related to protein synthesis, neuromuscular junctions maintenance, and fiber type switching (fast → slow) [35,63,64]. Corroborating to studies describing that besides anti-inflammatory properties, the glucocorticoids are also described by its ability to enhance mitochondrial biogenesis and to increase the proportion of type 1 muscle fibers [24], in which oxidative metabolism is predominant, with a significantly higher concentration of glucocorticoid receptor sites in the slow-fiber soleus muscle as compared with the fast-fiber extensor digitorum longus muscle [65].

Glucocorticoid administration induces an increase in *Myostatin* expression with a consequent increase in the protein and gene expression of Atrogin-1/MAFbx and MuRF-1 enzymes, mediated by FoxO3a and Smad2/3, leading to the start of the muscle atrophy process [19,47,66,67].

In our study, the expression of *Myostatin* was lower in groups receiving high doses of DX and DC (in comparison to lower doses), which may be related to a dose/time-dependence response of the exposed tissue to glucocorticoids [68,69]. All groups showed a significant increase of the *MURF-1* expression, except in the groups receiving DC. Significantly lower *Atrogin-1* expression was observed in the DC groups in comparison to the other glucocorticoid groups. These findings point to a milder effect on the expression of atrogenes by DC.

The expression of P-FOXO3a (a transcription factor responsible for the translation of the atrogenes *MuRF-1* and *Atrogin-1*) was similar between the groups. However, the gene and protein expression of atrogenes may show different results, as already described by Wang, et al. [70].

DC has been studied since the 1980s, and several factors have determined it as safer than other glucocorticoids. These findings are based on pharmacological properties, such as a lower excretion of calcium and hydroxyproline (amino acid present in collagen and abundant in the bone matrix), a lower effect on glucose metabolism and neuronal degeneration [71], lower effect on bone metabolism [72], and helping in preventing body fat accumulation and body growth retardation [73]. However, few studies have employed its use in healthy specimens evaluating muscle molecular pathways, and our study is more exploratory regarding this consideration. It is worth noting that DC is a compound that is mainly prescribed for the treatment of Duchenne’s muscular dystrophy (DMD) to bring benefic outcomes to treated patients. However, DC is associated with many side effects comparable to prednisolone/prednisone [74,75] and it was only released for use in the United States in February 2017 to treat DMD patients who are five years old or older (FDA note available at https://www.fda.gov/NewsEvents/Newsroom/PressAnnouncements/ucm540945.htm).

In our study the DC’s short-term effect on muscle atrophy, specifically in type 2B muscle fibers, and the lower atrogenes expression (*Atrogin-1/MAFbx* and *MuRF-1*), lead us to consider this compound as safer in a equipotent comparison between other glucocorticoids, despite the long-term effects, which can be similar or worse to other compounds, depending on the dose and days of administration.

Summarizing, we concluded that DC has a milder effect upon type 2B muscle atrophy and atrogenes expression, nevertheless, it boosts type 1 muscle fiber atrophy. Surprisingly, the lower doses of DC and DX are more harmful to slow-switch fibers CSA than the higher amounts.

MP interferes more with the body/peripheral muscles mass, myogenic program, muscle fiber type transition, and MEK/ERK pathway than the other glucocorticoids. In addition, DX is more harmful to the HPA axis and IGF-1 pathway (Akt/FoxO expressions) than DC and MP. Based on these assumptions, additional studies using equivalency will be useful to understand the action of glucocorticoids in the skeletal muscle as well as other tissues. This translational approach can drive to treatments more specific to the clinical goals, but considering other homeostatic processes that coexist.

## Figures and Tables

**Table 1:** Body mass average during the study.

Groups	Days of Study						
	1	30	32	34	36	38	40
CT	317.0 g	427.9 g	436.5 g	443.7 g	449.5 g	451.8 g	461.4 g
DX1.25	339.4 g	433.9 g	414.1 g	390.4 g ***	367.4 g ***	348.0 g ***	336.7 g ***
DX2.5	328.1 g	443.3 g	422.9 g	398.0 g **	374.6 g ***	357.1 g ***	335.5 g ***
DC10	317.5 g	418.2 g	380.2 g***	379.7 g ***	368.5 g ***	355.3 g ***	353.0 g ***
DC20	320.3 g	433.2 g	398.4 g **	385.0 g***	365.2 g ***	350.0 g ***	339.8 g ***
MP6	336.6 g	439.0 g	400.5 g *	368.5 g ***	346.0 g ***	323.4 g ***	317.0 g ***
MP13	319.1 g	436.0 g	388.1 g ***	354.8 g *** ## vs. DX2.5	331.4 g *** ## vs. DX2.5	313.1 g *** ## vs. DX2.5 # vs. DC20	293.4 g *** ## vs. DX2.5 ## vs. DC20

Asterisks represent the statistical analysis in comparison to the CT group or to the indicated group at each time of different glucocorticoids’ administration. * or # = *P* < 0.05; ** or ## = *P* < 0.01 and *** = *P* < 0.001 (*n* per group: CT = 10, DX1.25 = 9, DX2.5 = 8, DC10 = 10, DC20 = 10, MP13 = 8, MP6 = 8). A repeated-measures ANOVA, followed by the Bonferroni post hoc test, was used.

**Table 2:** Weight of collected adrenal and muscle tissues.

Group	Tissue Collected (Grams)		
	Adrenal	GA	TA
CT	0.0415 ± 0.007	2.398 ± 0.344	0.734 ± 0.103
DX1.25	0.0196 ± 0.002 ***	1.613 ± 0.257 ***	0.512 ± 0.038 ***
DX2.5	0.0208 ± 0.006 ***	1.774 ± 0.275 ***	0.477 ± 0.067 ***
MP6	0.0300 ± 0.012	1.570 ± 0.084 ***	0.500 ± 0.030 ***
MP13	0.0216 ± 0.003 ***	1.375 ± 0.088 ***; ## vs. DC20 and # vs. DX2.5	0.436 ± 0.043 ***; # vs. DC20
DC10	0.0311 ± 0.013	1.919 ± 0.205 *** and # vs. MP6	0.575 ± 0.053 ***; ## vs. MP6 and DX1.25
DC20	0.0227 ± 0.003 ***	1.801 ± 0.151 ***	0.549 ± 0.033 ***

Asterisks represent the statistical difference in comparison to the CT and (#) comparison between groups. # = *P* < 0.05; ## = *P* < 0.01 and *** = *P* < 0.001; (*n* per group: CT = 10, DX1.25 = 9, DX2.5 = 8, DC10 = 10, DC20 = 10, MP13 = 8, MP6 = 8).

**Table 3:** Muscle fiber type proportion after administration of different glucocorticoids. The Mann-Whitney test was used (*n* = 5 per group).

	Fiber 1	*P* Value	Fiber 2A	*P* Value	Fiber 2B	*P* Value
CT	12.83%	*P* = 0.0040 ** vs. MP6 *P* = 0.0213 * vs. MP13	27.17%	*P* = 0.0023 ** vs. DX2.5 *P* = 0.0180 * vs. DC10	60.00%	*P* = 0.0296 * vs. MP13
DX1.25	12.18%	-	26.50%	*P* = 0.0006 ** vs. DX2.5	61.32%	-
DX2.5	10.52%	-	34.56%	-	54.92%	-
MP6	5.60%	*P* = 0.0406 * vs. DX1.25 *P* = 0.0464* vs. DC10	30.66%	*P* = 0.0652 vs. MP13	63.74%	-
MP13	5.87%	-	25.00%	*P* = 0.0273 * vs. DC20 *P* = 0.0019 ** vs. DX2.5	69.13%	*P* = 0.0011 ** vs. DX2.5 *P* = 0.0010 ** vs. DC20
DC10	8.72%	-	32.60%	-	58.69%	-
DC20	9.54%	-	31.03%	-	59.43%	-

* = *P* < 0.05; ** = *P* < 0.01.
